# Explaining the effect on food selection of altering availability: two experimental studies on the role of relative preferences

**DOI:** 10.1186/s12889-022-13067-2

**Published:** 2022-04-30

**Authors:** Rachel Pechey, Gareth J. Hollands, Theresa M. Marteau

**Affiliations:** 1 Institute of Public Health, University of Cambridge, Cambridge, UK; 2 Nuffield Department of Primary Care Health Sciences, University of Oxford, Oxford, OX2 6GG UK; 3 EPPI-Centre, UCL Social Research Institute, University College London, London, UK

**Keywords:** Food, Availability, Mechanism, Preferences

## Abstract

**Background:**

Increasing the availability of healthier or plant-based foods increases their selection. The current studies aimed to examine the extent to which relative preferences account for food selections following availability interventions. In particular, (a) whether increasing the availability of lower-energy options increases the likelihood that individuals’ highest-ranked option is lower-energy, and (b) the extent to which selections reflect individuals’ highest-ranked option from the available range.

**Methods:**

UK adults (Study 1: *n* = 1976; Study 2: *n* = 1078) took part in within-subjects online studies. In both studies, the order of preference between food options was established by participants choosing the option that they would prefer “to eat right now” from every possible pairing within a pool of eight options. Then, participants were shown either predominantly higher-energy options (three higher- and one lower-energy) or predominantly lower-energy options (vice versa), presented in a random order.

**Results:**

When predominantly lower-energy options were presented, the odds of the highest-ranked option being a lower-energy option increased ten-fold (Study 1: odds ratio: 10.1; 95%CI: 8.9,11.4; Study 2: odds ratio: 10.4; 95%CI: 7.4,14.7), compared to when predominantly higher-energy options were available. In both studies, around 90% of selections reflected the highest-ranked option in the range offered in the studied availability conditions (range 88–92%).

**Conclusions:**

These studies suggest that increased availability of lower-energy options increases the likelihood of an individual’s highest-ranked option being lower-energy, and that the highest-ranked option has the greatest likelihood of selection. As such, preferences may be a key contributor to the effects of altering availability on food selections.

**Trial registration:**

ISRCTN (http://www.isrctn.com/ISRCTN27598623; 3/12/19 [Study 1]; http://www.isrctn.com/ISRCTN61010183; 20/4/20 [Study 2]).

**Supplementary Information:**

The online version contains supplementary material available at 10.1186/s12889-022-13067-2.

## Background

Increasing the availability of healthier snacks and main meals (e.g. [1, 2]) and plant-based meals [3] increases their selection [4]. A recent conceptual framework categorising availability interventions set out some of the potential mechanistic pathways that could underlie the effects of altering availability [5]. These mechanisms have been little explored, however. One of these potential pathways suggests that the effects of availability could be explained in terms of individuals tending to choose their most-preferred option in each instance – based on a mixture of their taste preferences from prior exposures [6] alongside their current needs and context [7, 8]. Other mechanisms, such as social norms regarding selection of different foods, may also act in parallel [9].

The impact of availability interventions could result from participants acting in line with their existing preferences. When options are added or removed the order of preference for each available product may change, including those not directly altered in the intervention. In particular, the type of food selected (healthier vs. less-healthy) may change if altering availability leads to a healthier option becoming the most-favoured option (over a less-healthy option), or dropping from this position. In addition, an individual’s order of preference between a set of options might change day-to-day – for example, choosing a more filling option when hungry [10] – and also adapt in response to new experiences of different options, e.g. positive associations resulting from consuming a particular food within an enjoyable context, or more negative associations following an unpleasant experience of a previously favoured food [11]. The degree to which the effects of availability could be explained by preferences may have implications for optimal implementation of any interventions.

Diet healthiness is socially patterned such that the poorest eat less-healthy diets [12, 13]. This contributes to the substantial socioeconomic inequalities in life expectancy and years lived in good health. As such, it is important that interventions targeting the availability of healthier foods do not differentially alter the food choices of those of higher socioeconomic position (SEP) relative to those of lower SEP, as this would exacerbate existing inequalities. As such, if the effects of availability are driven by individuals selecting their most-preferred option in each instance, increased healthier food availability might widen health inequalities if those with higher SEP are more likely to respond positively to healthier food cues. This is a potential concern given evidence of social patterning in food preferences, with higher SEP participants being more likely to favour healthier options (e.g. [14, 15]). Patterning observed in some studies has been consistent with potential differential impact of availability interventions by SEP, suggesting those with higher SEP may be more likely to respond to increased healthier food availability, but with insufficient power to test effects [2, 16]. Establishing the mechanisms that might underlie the impact of availability may help establish how best to implement this promising intervention [17].

The current set of studies aimed to provide the first test – to our knowledge – of the role of relative preferences as a possible mechanism underlying the effects on selection of manipulating the relative availability of healthier food options. In particular, we focus on lower-energy vs. higher-energy food options, representing one dimension of food healthiness, to test the following hypotheses:

1. Increasing the relative availability of lower-energy options increases the likelihood that an individual’s highest-ranked option is a lower-energy option
2. The option selected by an individual tends to reflect their highest-ranked option from the possible range of options available

In addition, we extended Hypothesis 1 to suggest that increasing the relative availability of lower-energy options increases the likelihood that an individual’s highest-ranked option is a lower-energy option to a greater extent for those with higher (vs. lower) SEP (Hypothesis 1a).

## Methods

This paper reports on two studies; the first part of both aimed to establish an order of preference between available options. This was done by asking participants to choose which of a pair of options they would like “to eat right now”. The order of preference was established without explicitly asking participants to rate their preferences, given that explicit ratings of preferences might reflect a more general pattern of preferences over time than relative preferences at the point in time studied [7] – as well as the risk that drawing attention to preferences might sway selections to correspond to these stated preferences. Then in the second part of both studies, the impact of varying the relative availability of higher- vs. lower-energy options on the likelihood that participants’ highest-ranked option is a lower-energy option was examined.

Study 2 was a replication of Study 1. This study aimed to extend the results of Study 1 by including options for which there is a larger discrepancy in preferences between the lower- vs. higher- energy foods, as observed in a pilot study. (The pilot study sample was representative of the UK by age and gender, and included quotas ensuring an even distribution by highest educational qualification.) Study 2 allowed testing of the robustness of results across different scenarios. In particular, it examined first whether increasing the number of a set of food options known to be less-preferred by a population group in general (e.g. lower-energy meals), can increase uptake of these options, and second, whether this uptake can still be explained in terms of the preferences of the individuals involved.

Studies were pre-registered on the Open Science Framework (https://osf.io/hz9t5 [Study 1]; https://osf.io/yjmpe [Study 2]) and ISRCTN (http://www.isrctn.com/ISRCTN27598623 [Study 1]; http://www.isrctn.com/ISRCTN61010183 [Study 2]). Ethical approval was obtained from the University of Cambridge Psychology Research Ethics Committee (Refs: PRE.2019.087 [Study 1]; Pre.2020.030 [Study 2]).

### Participants

For both studies, a sample of UK adults was recruited from a market research agency panel, with quotas set by education to obtain equal numbers by highest educational qualification (Lower: Up to GCSE level or 1 A Level; Higher: 2 + A Levels or equivalent, or higher qualification [GCSEs (General Certificate of Secondary Education) are usually taken at around age 16, A-levels are typically taken at around age 18 in the UK, and represent qualifications that would be recognised as entry requirements to higher education]). For Study 2 additional quotas were used to ensure a representative sample by age and gender. Individuals who self-reported having any dietary restrictions (e.g. vegetarians) were excluded from both studies, to ensure that participants felt they had a choice between the options offered. Participants who failed attention check questions were excluded (*n* = 165 in Study 1; *n* = 210 in Study 2), as was anyone completing the studies in less than 30% of the median time for that study (one participant in Study 1, none in Study 2). Participants who completed Study 1 were not eligible for Study 2.

#### Sample size Study 1

The sample size was determined using a simulation-based approach to predicting power for a multilevel logistic regression [18]. The calculation was based on 100 replications, for a model with: four level 1 units (representing the four selections made by each participant) and three binary covariates: two at level 1 (availability condition and food type) and one at level 2 (education). The calculation assumed each of these groups had equal numbers of participants. For a conservative model where each of the covariates had a small effect size (Cohen’s d of 0.2), and the differences between individuals were relatively large (intercept variance of 1.5), simulations suggested that a sample of 1950 individuals would achieve a power of 0.8 or above for estimates of each of the covariates.

#### Sample size Study 2

As above, the sample size was determined using a simulation-based approach, for a multilevel logistic regression [18], based on 100 replications. This was calculated for a model with: two level 1 units (representing the two selections made by each participant), and one level 1 binary covariate – the availability condition [d = 0.38, equivalent to the smallest effect of availability found in previous online studies; participants distributed evenly between groups]. The intercept variance was assumed to be 0.64 and beta 1.4 [from Study 1]. Simulations suggested that a sample of 1080 individuals would achieve a power of 0.9 for the estimated effect of availability on whether or not participants’ pre-existing most-preferred option was lower-energy.

### Design

Both Study 1 and Study 2 were conducted online using a within-subjects design, comparing choices between food options from ranges of options comprised of (a) one lower-energy, three higher-energy options; (b) three lower-energy, one higher-energy options. Four options were selected for these choice sets based on the standard number of options observed in cafeteria offerings in previous studies [2, 19].

For Study 1, two sets of food type options were investigated: (i) branded snack items and (ii) unbranded main meal options, given existing preferences may be a stronger influence for branded snacks – with known taste – compared with unbranded main meals – with unknown taste. Study 2 focused on main meal options only.

### Measures and materials

#### Food options

##### Study 1

Eight options were identified for each of the branded snack and unbranded main meal conditions: four classed as lower-energy and four as higher-energy options. Energy content represents one component contributing to diet healthiness. Excess energy intake contributes to overweight and obesity, which in turn are associated with type 2 diabetes and certain cancers [20, 21]. This was selected as a readily available proxy for healthiness, to test whether preferences might act as a mechanism underlying availability interventions.

**Fig. 1:**
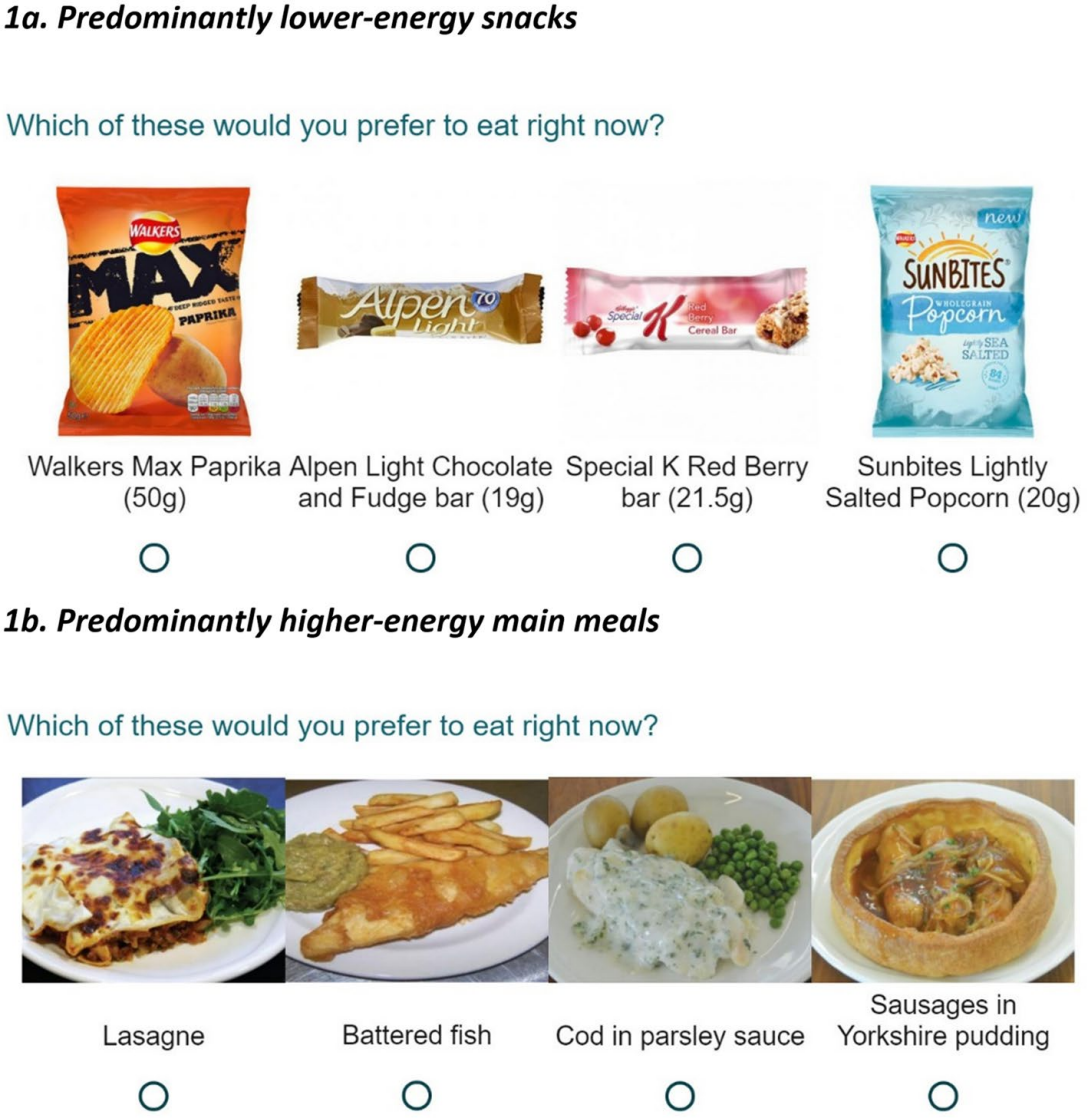
Examples of option sets shown to participants with varying availability of lower-energy options. **a** Predominantly lower-energy snacks. **b** Predominantly higher-energy main meals. N.B. Other snack options presented were: Higher-energy: Lindt Milk Chocolate Orange bar (38 g), Niknaks Nice ‘n’ Spicy (50 g), Reese’s Snack Mix (56 g); Lower-energy: Walkers Pops Original (19 g); see Table 1 for other main meal options

For branded snacks, lower-energy options were defined as 100 kcal or less per pack, and higher-energy 200 kcal or more per pack (as in Pechey & Marteau, 2018). Pictures of branded snack options were taken from those used in previous online studies [22], where pilot work has established that their perceived healthiness was in line with the above categorisation, these were matched in terms of familiarity, and represent a single serving. Picture descriptions included the weight of pre-packaged products (see Fig. 1a for an example question).

Main meals were considered lower-energy if they were under 500 kcal for a complete meal, and higher-energy if they were 500 kcal or more (as in Pechey et al., 2019). Pictures of unbranded main meals were taken from a manual used by worksite cafeterias for a major supermarket chain (see Table 1 for the images used in both studies). These pictures showed meals made in these cafeterias (in the portion sizes served), and their energy (kcal) content was provided in the manual. To ensure that the energy content of the meals pictured was in line with that expected for this meal, three alternative recipes for each meal were found, and it was checked that the energy content of our pictured meal fell within this range.

**Table 1:**
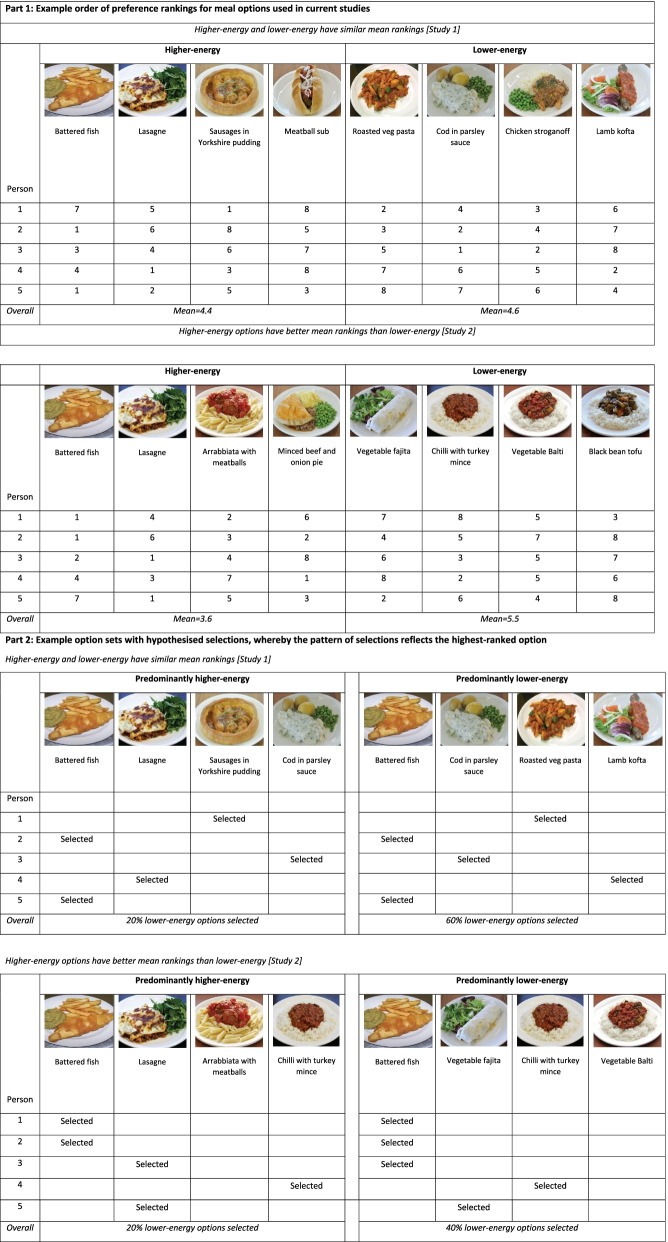
Part 1: Example order of preference rankings for meal options used in current studies. Part 2: Example option sets with hypothesised selections, whereby the pattern of selections reflects the highest-ranked option

##### Study 2

As above, eight main meal options were used, with four classed as lower-energy and four as higher-energy options. Options met the same definition for lower vs. higher energy, and were taken from the same manual. In order to select higher vs. lower energy options which differed in terms of relative preference, options were chosen from the results of a pilot study (540 participants), in which the most-preferred four higher-energy options, and least-preferred four lower-energy options were selected.

#### Socioeconomic position

The primary measure used for both studies was highest educational qualification, subdivided into two groups: higher (degree or above) vs. lower (up to GCSE-level education or equivalent). Annual household income was collected as an additional measure of SEP. For Study 1 only, occupational group (A&B: Higher and intermediate managerial, administrative and professional occupations; C1&C2: Supervisory, clerical and junior managerial, administrative and professional occupations; D&E: Semi-skilled and unskilled manual occupations) was also collected.

#### Other demographic characteristics

Ethnicity, hunger (self-reported on a 7-pt scale from “Very hungry” to “Very full”), and height and weight (to calculate body mass index) were also collected to provide sample demographic characteristics.

### Procedure

Both studies were completed online using Qualtrics. The studies followed the same procedure, but participants completed twice as many trials in Study 1, which included both snacks and main meal food options, whereas Study 2 only examined the latter.

#### Part 1: Establishing an order of preference between options

During the first part of each study participants were presented with pictures of two food options, and asked to choose which they would “prefer to eat right now”. They completed this task for every possible item pair (*n* = 28), each of which was presented twice so as to collect more than one data point for each pair (56 trials). For Study 1, this was done for images of snacks and main meals, giving a total of 112 trials (vs. 56 trials in Study 2).

Responses to these trials were used to calculate participants’ order of preference between items. For each trial, the selected item received a score of 1. Scores were summed across all trials for each item. Order of preference rankings for the available options were created for each participant, from 1 (most-selected from paired-selections) to 8 (least-selected). For ties, both tied items’ rankings were recorded as 1.5, 2.5 or 3.5 (i.e. tied for first, second or third place respectively). Separate scores were calculated using this method for snacks and main meals in Study 1.

#### Part 2: Impact on selection of varying availability

In the second part of the studies participants were shown a series of pictures depicting a set of options (see Fig. 1). For each set participants were asked to select which option they would “prefer to eat right now”.

For Study 1, four option sets were presented to each participant: (1) Predominantly-higher-energy branded snacks, (2) Predominantly-lower-energy branded snacks, (3) Predominantly-higher-energy unbranded main meals, and (4) Predominantly-lower-energy unbranded main meals. For Study 2, only the latter two option sets were examined. The order in which these sets were presented to participants was randomised.

To ensure that results did not depend on the presence of particular options within a set of food, the eight possible food options were randomised to one of the two availability conditions, such that the Predominantly higher-energy set of food options had one lower-energy and three higher-energy options and the Predominantly lower-energy had three lower-energy and one higher-energy options. As such, the foods comprising each set varied between participants. Options within each set were also randomised to their position in the display (far right, middle right, middle left, far left).

Participants then completed measures on age, gender, educational qualifications, household income, ethnicity, height, weight and hunger.

### Analyses

Analyses followed the same procedure for both Study 1 and Study 2.

#### Manipulation checks

1. Difference in preferences between lower-energy vs. higher-energy options: Wilcoxon signed-rank tests were used to test whether higher-energy options were preferred over lower-energy options in each study, with separate tests for branded snacks and unbranded meals in Study 1.
2. Impact of availability on selection of lower-energy options: Analysed using a mixed effects logistic regression, conducted at the participant-level, comparing whether ranges of options of (a) one lower-energy, three higher-energy options or (b) three lower-energy, one higher-energy options for (i) branded snack or (ii) unbranded main meal options alter the likelihood of participants’ selecting lower-energy options, with random effects for participant. Covariates included age, gender, and hunger.

#### Hypothesis 1

The primary outcome was whether participants’ highest-ranked option was a lower-energy option (vs. higher-energy option). The analysis used mixed effects logistic regression, conducted at the participant-level, comparing whether offering ranges of options containing (a) one lower-energy, three higher-energy options vs. (b) three lower-energy, one higher-energy options, alter the likelihood that participants’ highest-ranked option is a lower-energy option, with random effects for participant. Covariates were age, gender and hunger.

#### Hypothesis 2

The primary outcome was the correspondence between participants’ selection and their highest-ranked option (coded as ‘1’ for a match; ‘0’ otherwise). This was assessed via descriptive statistics of the proportion of selected options that were ranked 1^st^, 2^nd^, 3^rd^ or 4^th^ according to participants’ order of preference rankings within the offered choice set.

Ties: Given analyses were conditional on determining whether participants’ highest-ranked option was lower or higher-energy, any option sets where a lower-energy and a higher-energy option were tied for first place (i.e. highest-ranked option) were excluded from analyses.

#### Hypothesis 1a and Secondary Research Questions

Hypothesis 1a included SEP as a potential moderator in the model used for Hypothesis 1. Secondary research questions explored two other potential moderators of the above analyses: (1) whether the option was lower or higher energy, and (2) food type. For these analyses, all trials for which the highest-ranked option was unable to be established (due to there being a tie for first place) were excluded. See supplementary materials for detailed analytic plan for these questions.

For our main analyses, we used *p* < 0.05 (two-tailed) to infer if there was a statistically significant effect. For the Secondary Research Questions, we used a Bonferroni correction to account for the different hypotheses tested, taking a *p*-value < 0.003 (two-tailed) for Study 1 (*p* = 0.05/15), and *p* < 0.004 (two-tailed) for Study 2 (*p* = 0.05/12).

## Results

### Participant characteristics

In Study 1, a total of 1976 participants completed the survey (see Table 2), 51% of whom were female, with 50% having higher and 50% lower education. Participants were older than the UK population average (mean age of 61 years). Of the 1078 participants in Study 2, 50% were female, with again an even split achieved by education. Participants’ mean age was 47 years.

**Table 2:** Characteristics of participants in each study

		**Study 1**	**Study 2**
Gender [% (n)]	Male	48.7 (963)	50.2 (541)
	Female	51.2 (1011)	49.8 (537)
	Other	0.1 (2)	0 (0)
Age	*Mean (s.d.)*	61.4 (11.4) ^a^	47.3 (16.9)
Education [% (n)]	Lower (Up to 1 A Level)	49.7 (982)	50.1 (540)
	Higher (2 A Levels or higher)	50.3 (994)	49.9 (538)
Income [% (n)]	Up to £17,499	24.1 (476)	23.9 (258)
	£17,500-£29,999	26.2 (518)	21.2 (228)
	£30,000-£49,999	26.6 (526)	27.6 (297)
	£50,000 +	17.2 (339)	20.2 (218)
	Prefer not to say / missing	5.9 (117)	7.1 (77)
Occupational group^b^ [% (n)]	A&B	35.3 (697)	-
	C1&C2	42.4 (837)	-
	D&E	22.1 (436)	-
	Missing	0.3 (6)	-
Ethnic group [% (n)]	White	95.9 (1894)	94.1 (1014)
	Other	2.8 (56)	5.7 (61)
	Missing	1.3 (26)	0.3 (3)
BMI group [% (n)]	Under 25	36.0 (711)	41.9 (452)
	25–30	35.0 (692)	28.2 (304)
	30 +	20.6 (407)	20.1 (217)
	Missing	8.4 (166)	9.7 (105)
Hunger^c^	*Mean (s.d.)*	0.31 (1.28) ^a^	0.50 (1.30)
Total participants	*N*	1976	1078

^a^ Of the 1976 participants in Study 1, eight did not report age and six did not report hunger

^b^Occupational group was not collected in Study 2. A&B: higher managerial and professional; C1&C2: white collar and skilled manual; and D&E: semi-skilled and unskilled manual

^c^Hunger was self-reported on a 7-pt scale from “Very hungry” (3) to “Very full” (-3)

### Manipulation checks

#### Difference in preferences between lower-energy vs. higher-energy options.

##### Study 1

For snacks, lower-energy items (mean ranking 4.39, s.d. 0.98; rankings go from 1 (most-selected) to 8 (least-selected)) were preferred to higher-energy ones (mean ranking 4.61, s.d. 0.98; Wilcoxon signed rank test Z = -4.733, *p* < 0.0001). For main meals, higher-energy options (mean ranking 4.37, s.d. 0.95) were preferred to lower-energy ones (mean ranking 4,63, s.d. 0.95; Wilcoxon signed rank test Z = 6.245, *p* < 0.0001). Overall differences were small.

##### Study 2

The differences in preferences were greater than in Study 1 (as expected, given items were selected to differ according to this variable), with higher-energy meals (mean ranking 3.50, s.d. 0.96) being preferred to lower-energy ones (mean ranking 5.50, s.d. 0.96; Wilcoxon signed rank test Z = 23.512, *p* < 0.0001).

#### Impact of availability on selection of lower-energy options

##### Study 1

**Fig. 2:**
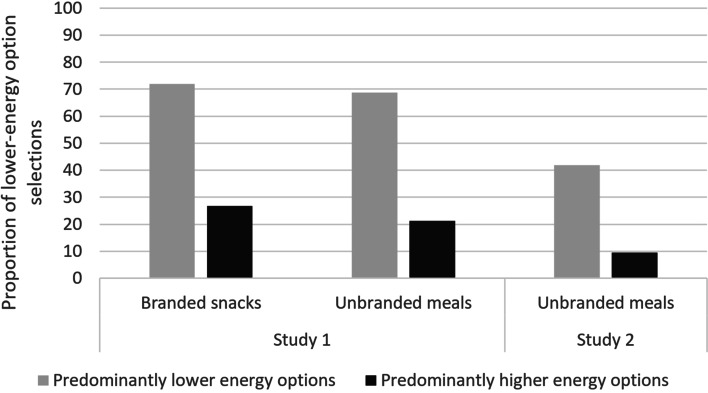
Proportion of lower-energy option selections by availability condition, food type and study

Overall, 26.8% (*n* = 529) of participants selected a lower-energy snack when there were predominantly higher-energy snacks available, rising to 72.0% (*n* = 1423) when there were predominantly lower-energy snacks available. For main meals, 21.2% (*n* = 419) of participants selected a lower-energy meal when the set contained predominantly higher-energy options and 68.8% (*n* = 1359) when the set contained predominantly lower-energy options (see Fig. 2).

##### Study 2

When options were predominantly higher-energy, 9.6% (*n* = 103) of participants selected a lower-energy meal, rising to 41.9% (*n* = 452) when options were predominantly lower-energy.

The Table 1 illustrates these patterns of results, highlighting that even if on average lower-energy options might be less preferred to higher-energy ones (e.g. [23]), increasing the availability of lower-energy options could still be expected to increase lower-energy selections in line with individuals’ preferences. Note that the proportions selected following the availability manipulations are similar to those predicted in the Table 1 (Part 2); the preference rankings in Part 1 of the Table 1 were based on those found in Study 1 and 2.

Logistic regressions: Having predominantly lower-energy options increased the odds of selecting a lower-energy option, with odds ratios of 8.9 (95%CI: 7.9, 10.1) in Study 1 and 9.7 (95%CI: 7.0, 13.5) in Study 2, compared to predominantly higher-energy options. See Supplementary tables S1a and S1b for full regression results for each study respectively.

### Hypothesis 1: Increasing the relative availability of lower-energy options increases the likelihood that an individual’s highest-ranked option is a lower-energy option

**Fig. 3:**
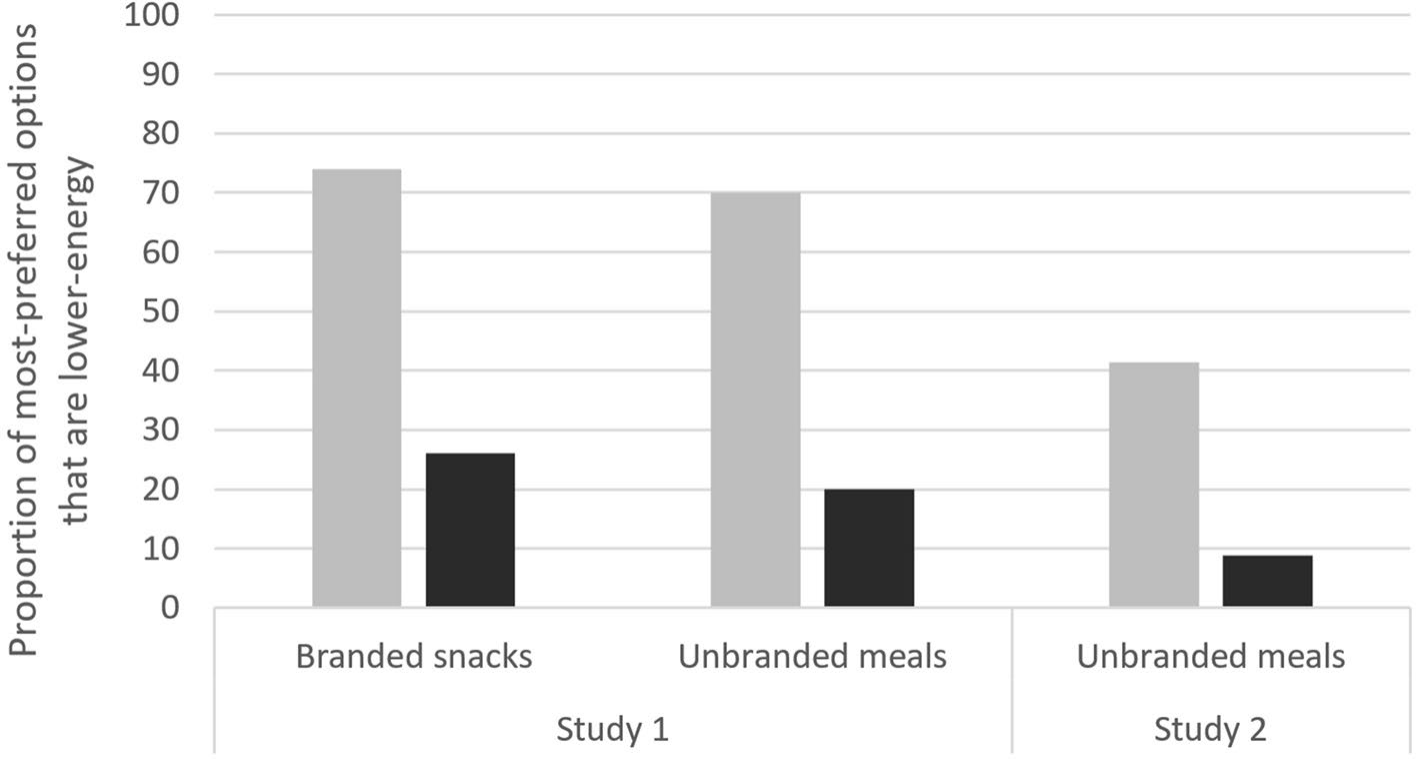
Proportion of highest-ranked options that are lower-energy, by food type and study. N.B. Excludes trials with tied highest-ranked options that were lower-energy and higher-energy

In general, the pattern of results by study and food type when examining the proportion of highest-ranked options that were lower-energy was very similar to the patterning seen for selection of lower-energy options (see Fig. 3).

#### Study 1

The highest-ranked option was a lower-energy option for 26% (*n* = 500) of participants when there were predominantly higher-energy snacks available, rising to 74% (*n* = 1404) when there were predominantly lower-energy snacks available. For main meals the highest-ranked option was a lower-energy option for 20% (*n* = 380) of participants when the set contained predominantly higher-energy options and for 70% (*n* = 1336) when the set contained predominantly lower-energy options.

#### Study 2

The highest-ranked option was a lower-energy meal for 9% (*n* = 93) of participants when the meals available were predominantly higher-energy, compared to 41% (*n* = 429) when the meals available were predominantly lower-energy.

Logistic regressions: When predominantly lower-energy options were available, the odds of the highest-ranked option being a lower-energy option increased ten-fold (Study 1: odds ratio: 10.1; 95%CI: 8.9, 11.4; Study 2: odds ratio: 10.4; 95%CI: 7.4, 14.7), compared to predominantly higher-energy options being available. See Supplementary tables S2a and S2b for full regression results for each study respectively.

### Hypothesis 2: The option selected by an individual tends to reflect their highest-ranked option from the possible range of options available

Table 3 shows that around 90% of selections reflect the highest-ranked option in the range offered in each of the availability conditions – and by each food type – in Study 1 and Study 2 (range 88–92%). Of those selections that do not involve the highest-ranked option, the majority of selections then reflect the second highest-ranked option (7–10% of the overall trials).

**Table 3:** Proportion of selections by order of preference ranking, availability condition, food type and study

			Proportion of option selections (% (n))			
	Order of preference ranking for option within range offered^a^		1 (Most preferred)	2	3	4 (Least preferred)
Study 1	Branded snacks	Predominantly lower-energy availability	88.2 (1743)	10.2 (201)	1.4 (28)	0.2 (4)
		Predominantly higher-energy availability	89.4 (1766)	9.5 (188)	1.0 (19)	0.2 (3)
	Unbranded meals	Predominantly lower-energy availability	91.6 (1810)	7.4 (147)	0.7 (12)	0.3 (5)
		Predominantly higher-energy availability	91.1 (1800)	7.6 (151)	1.1 (22)	0.1 (2)
Study 2	Unbranded meals	Predominantly lower-energy availability	88.5 (954)	9.1 (98)	1.6 (17)	0.8 (9)
		Predominantly higher-energy availability	87.6 (944)	9.2 (99)	2.1 (23)	1.7 (18)

^a^Ties (e.g. 1.5) rounded down (i.e. 1.5 to 1)

### Hypothesis 1a

**Fig. 4:**
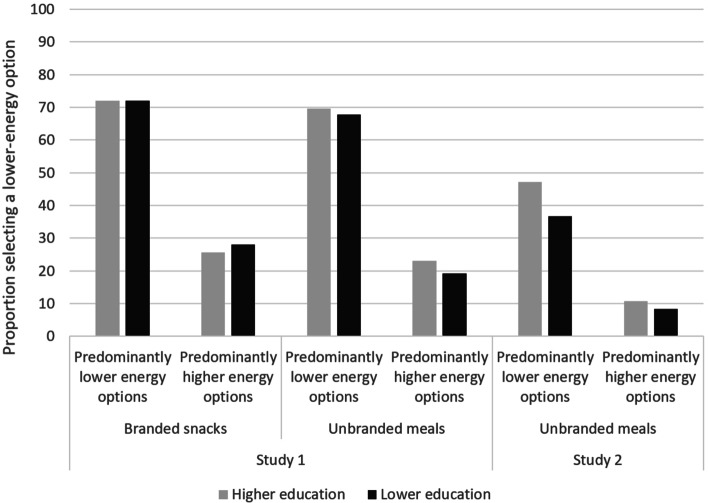
Proportion selecting a lower-energy option by level of education. See Supplementary Table S10a for number of observations in each group

Figure 4 shows the patterning in selection of lower-energy options by education. In Study 1, this did not suggest differences by education for either snacks or meal selection. In Study 2, however, those with higher education were more likely to select a lower-energy meal option (11% [*n* = 58] with predominantly higher-energy availability; 47% [*n* = 254] when predominantly lower-energy; compared to 8% [*n* = 45] and 37% [*n* = 198] respectively for those with lower education).

There was no moderation of the impact of availability on selection by education in either study (Study 1: main effect: odds ratio: 1.1; 95%CIs: 0.9, 1.3; *p* = 0.222; interaction term: odds ratio: 1.0; 95%CIs: 0.8,1.2; *p* = 0.893; Study 2: main effect: odds ratio: 1.4; 95%CIs: 0.9, 2.1; *p* = 0.189; interaction term: odds ratio: 1.3; 95%CIs: 0.8,2.1; *p* = 0.381; see Supplementary tables S6a and S6b). Similarly, no moderation of the availability manipulation was found for additional socioeconomic indicators (household income and occupational group; see Supplementary tables S7a, S7b & S8a). In accordance with the analysis plan, further modelling looking at moderation of either Hypothesis 1 or Hypothesis 2 was not conducted. Supplementary Tables S10a-S10d provide descriptives for selections, rankings, and highest-ranked options by education.

### Secondary research questions

#### Energy content of highest ranked option

Hypothesis 2: In Study 1, if the highest-ranked option was lower-energy, it was less likely – with an odds ratio of 0.80 (95%CIs: 0.67, 0.95; *p* = 0.013) – that the selected option matched that highest-ranked option, although this did not reach statistical significance at the pre-specified value of *p* < 0.003.

A similar pattern was found in Study 2, with a lower-energy highest-ranked option being less likely to match the selected option than a higher-energy highest-ranked option, with an odds ratio of 0.27 (95% CIs: 0.17, 0.42; *p* < 0.001) (See Supplementary tables S4a and S4b for full model results; Table S9 for the breakdown by each ranking position).

#### Food type (Study 1)

##### Hypothesis 1

Participants’ highest-ranked option was more likely to be lower-energy for snacks than main meals (odds ratio: 1.3; 95%CIs: 1.2, 1.5; *p* < 0.001). Adding an interaction between food type and availability condition into the Hypothesis 1 model did not suggest any difference in the likelihood of participants’ selected option matching their highest-ranked option by food type (main effects of snacks (over main meals): odds ratio: 1.5; 95%CIs: 1.2, 1.7; *p* < 0.001; interaction term: odds ratio: 0.8; 95%CIs: 0.7,1.0; *p* = 0.123). See Supplementary table S3 for full model.

##### Hypothesis 2

Participants’ highest-ranked snack options were less likely to match their selected items than main meal options (odds ratio: 0.7; 95%CIs: 0.6, 0.9; *p* < 0.001; see Supplementary table S4a).

## Discussion

Both studies suggested that participants’ highest-ranked option is more likely to be a lower-energy option when lower-energy options are increased, and around 90% of selections reflected the highest-ranked option, regardless of the range offered. This provides the first evidence of the extent to which relative preferences might underlie the impact of availability interventions. Given that both studies showed similar results, this suggests that the changes in option selection after implementation of an availability intervention may largely be driven by individual preferences. Moreover, the results of Study 2 suggest that the impact of increasing the availability of lower-energy options may still be accounted for primarily through individual preferences even if higher-energy foods are on average preferred to lower-energy ones.

Both studies showed a large effect of availability, similar to previous results in a recent online study [22] in which the odds of selecting a lower-energy option increased almost ninefold when options were predominantly healthier, compared to predominantly less-healthy, similar to nearly ninefold and tenfold increases in odds in the current studies. This is also consistent with field studies suggesting that increasing the availability of lower-energy (e.g. [1, 2]) and plant-based [3] foods increases their selection. The absolute levels of lower-energy option selection varied between Study 1 and 2, with 21% and 12% respectively selecting a lower-energy option when there were predominantly higher-energy options, which approximately tripled when there were predominantly lower-energy ones. This likely reflects the larger discrepancy on average in preferences between lower-energy and higher-energy options in Study 2.

The pattern for the proportion of highest-ranked options that were lower-energy was very similar to the patterning seen for selection of lower-energy options, including in terms of the differences between studies – with the proportion of highest-ranked options that were lower-energy being lower in Study 2 than in Study 1. However, the impact of having predominantly lower-energy options, compared to predominantly higher-energy options on the odds of the highest-ranked option being a lower-energy option were just over tenfold in both of the studies. This suggests that while the discrepancy in average preferences between lower-energy and higher-energy options impacted on absolute likelihood of an individual’s highest-ranked option being a lower-energy option, it did not change the relative likelihood that the highest-ranked option became lower-energy when availability was altered.

The studies suggested that between 88–92% of selections reflected the highest-ranked option in the range offered in each of the availability conditions. As would be expected, if selections did not match the highest-ranked option, the majority then reflected the second highest-ranked option. Together with the above explorations of the energy content of the highest-ranked options under each availability condition, this offers evidence supporting both study hypotheses.

Explorations of potential moderators suggested that a lower-energy highest-ranked option was less likely to match the selected option than a higher-energy highest-ranked option in Study 2, but this was a smaller effect and not statistically significant in Study 1. This could indicate that the preferences for these lower-energy options tended to be less distinct (i.e. the highest-ranked option was closer in terms of preferences to the second highest-ranked) in Study 2, leading to greater fluctuations between selection of options. This could be explored in future research.

In terms of comparing snacks vs. main meals, there was no evidence of a difference in the likelihood of participants’ selected option matching their highest-ranked option by food type, but participants’ highest-ranked snack options were less likely to match their selected items than main meal options. This is in contrast to our prediction that existing preferences may be a stronger influence for branded snacks compared with unbranded main meals. It is possible that the distinction between lower-energy and higher-energy options was more easily discernible for snacks than for main meals, possibly leading to social desirability effects towards the end of the study. Alternatively, it may be that preferences for snacks, typically eaten more quickly and being less substantial than a meal, may be more easily overridden. Such effects – if replicated – will require further research to tease apart.

No moderation of the impact of availability on selection of lower-energy options was found by education in either study. In Study 2 – but not Study 1 – those with higher education were more likely than those with lower education to select a lower-energy option (11% vs. 8% for predominantly higher-energy availability; 47% vs. 37% for predominantly lower-energy), with a near-identical pattern of results for their highest-ranked options. This suggests that while there may be differences in preferences by education in some contexts, the effects of these relative availability interventions were equitable across different levels of educational attainment. This is similar to the differences in the absolute but not relative effects of availability observed between Study 1 and Study 2 – i.e. the proportional changes were similar across studies, but the absolute differences were altered in line with the proportion selecting lower-energy options across availability conditions (higher in Study 1). However, it is possible that differences in preferences by education may make some availability interventions more likely to be effective in higher education groups (e.g. increasing lower-energy food availability without simultaneously reducing higher-energy food availability). This should be addressed in future research.

### Strengths and limitations

This set of studies offers a novel examination of the potential role of preferences in underlying the impact of altering the availability of options in order to change dietary behaviour. By showing similar results in two large studies, designed to reflect situations with varying degrees of discrepancy between average preferences for target (e.g. lower-energy) and non-target (e.g. higher-energy) options, these results suggest the robustness of the role of preferences. Similarly, finding a consistent pattern of results for both branded snacks and unbranded main meals adds to the potential generalisability of these findings to different food contexts. In addition, the inclusion of explorations by SEP allow some evaluation of the possible impact on socioeconomic inequalities in response to the types of availability interventions used in the current studies.

Key limitations, however, include that these were online studies, in which participants did not receive the foods they selected, limiting ecological validity. The focus of the current studies was to explore mechanisms, however, rather than real-world generalisability. Indeed, in a real-world scenario, individuals can choose not to select any of the available options – particularly if generally less appealing options are chosen for the lower-energy category as in Study 2 – whereas in the current studies, participants were obliged to select one. The methods used here to establish order of preference only indicate which items are preferred relative to each other; and assume these are relatively internally consistent (i.e. if A is preferred to B, and B to C, then A would be preferred to C). Finally, we have discussed the differences between studies primarily in terms of the differences established by design (i.e. a greater discrepancy on average in preference between target and non-target options), but other differences may also contribute, including the different samples and nature of the individual food options included in the studies, such as the perceived healthiness of the higher vs. lower-energy meal options, which we did not assess.

### Implications for research and policy

This set of studies offers a first exploration of the role of preferences in the context of relative availability interventions. It opens up a series of possible future research questions that could be explored – including further investigation of the moderators of the effects that were identified in this paper, to identify more clearly the impacts these might have in different contexts. In addition, given the results of this paper suggest that preferences may indeed be playing a key role, their influence on different kinds of availability interventions [5] – for example, only adding in lower-energy options, or only removing higher-energy ones – would be interesting to determine, and particularly, whether such interventions would show any socioeconomic differences in their impact.

If, as suggested in these results, availability interventions are dependent on underlying preferences, this could have implications for how availability should be altered to target behaviour change. For example, interventions may need to remove more favoured higher-energy options, as if lower-energy options are less preferred, interventions that focus only on adding lower-energy options may be less effective. Indeed, if these results are supported further, the selection of which items to target to maximise intervention effectiveness could take into account preferences – with the caveat that an option that is acceptable to individuals would still need to be available if it is desirable to avoid people selecting no option at all. In these studies, while there were some differences in preferences by SEP (in Study 2), the effects of the relative availability interventions, altering both higher- and lower-energy options, were equitable across socioeconomic groups. As such, if reproduced, these interventions would not have widened – or reduced – any existing inequalities in dietary selections. Availability interventions may prove a useful tool for equitable dietary change, and may be enhanced if complementary measures can be implemented to shift food preferences particularly in lower SEP groups.

Preferences are determined by a range of factors, for example, individuals’ preference for high-fat food may be linked to their physiological responses and perceptions [24], which interact with their social context and personal experiences at the time of food choice [25]. Targeting preferences themselves may increase lower-energy food selection, but potential interactions with other intervention strategies such as availability may increase the effectiveness of both. For example, creating positive associations with lower-energy foods, e.g. through marketing, may help to improve relative preference for lower-energy compared to higher-energy options [26]. Furthermore, if availability interventions increase lower-energy option selection, this may in turn increase preferences for these options through their increased exposure [27].

## Conclusions

This set of studies aimed to test two hypotheses: (1) Increasing the relative availability of lower-energy options increases the likelihood that an individual’s highest-ranked option is a lower-energy option; (2) The option selected by an individual tends to reflect their highest-ranked option from the possible range of options available. Evidence found offered strong support for both hypotheses, suggesting the key role of preferences as a contributory factor underlying the impact of altering the availability of options on food selection. This could have implications for how availability interventions might be optimally implemented to ensure such interventions are effective, and in particular, effective for those for whom behaviour change is most beneficial.

## Supplementary Information

**Additional file 1.**

## Data Availability

The datasets generated during this set of studies are available in the Open Science Framework (Study 1: https://osf.io/f3thq/; Study 2: https://osf.io/yj89x/; data dictionary: https://osf.io/m7zfr/).

## Abbreviation

SEP: Socioeconomic position
