# Supplementary material for: Explaining the effect on food selection of altering availability: two experimental studies on the role of relative preferences
Source: BMC Public Health. 2022 Apr 30;22:868. doi: 10.1186/s12889-022-13067-2 (PMC9063226; doi:10.1186/s12889-022-13067-2)
Supplement: Supplementary file 1 — Additional file 1. [file 12889_2022_13067_MOESM1_ESM.docx]

**Supplementary materials**

Contents

[Analysis Plan: Secondary Research Questions 3](#_Toc87386382)

[Table S1a. Manipulation check, Study 1: Mixed effects logistic regression results predicting whether participants selected a lower-energy option. 4](#_Toc87386383)

[Table S1b. Manipulation check, Study 2b: Mixed effects logistic regression results predicting whether participants selected a lower-energy option. 4](#_Toc87386384)

[Table S2a. Research Question 1, Study 1: Mixed effects logistic regression results predicting whether participants’ highest-ranked option was a lower-energy option. 5](#_Toc87386385)

[Table S2b. Research Question 1, Study 2b: Mixed effects logistic regression results predicting whether participants’ highest-ranked option was a lower-energy option 5](#_Toc87386386)

[Table S3. Secondary Research Question 3a, Study 1: Mixed effects logistic regression results predicting whether participants’ highest-ranked option was a lower-energy option. 6](#_Toc87386387)

[Table S4a. Secondary Research Question 4a, Study 1: Mixed effects logistic regression results predicting whether participants’ selected option corresponds to their highest-ranked option. 7](#_Toc87386388)

[Table S4b. Secondary Research Question 4a, Study 2b: Mixed effects logistic regression results predicting whether participants’ selected option corresponds to their highest-ranked option. 7](#_Toc87386389)

[Table S6a. Secondary Research Question, Manipulation Check, Study 1: Mixed effects logistic regression results predicting whether participants selected a lower-energy option. 8](#_Toc87386390)

[Table S6b. Secondary Research Question, Manipulation Check, Study 2b: Mixed effects logistic regression results predicting whether participants selected a lower-energy option. 9](#_Toc87386391)

[Table S7a. Income Group Analysis; Secondary Research Question, Manipulation Check, Study 1: Mixed effects logistic regression results predicting whether participants selected a lower-energy option. 10](#_Toc87386392)

[Table S7b. Income Group Analysis; Secondary Research Question, Manipulation Check, Study 2b: Mixed effects logistic regression results predicting whether participants selected a lower-energy option. 11](#_Toc87386393)

[Table S8a. Occupational Group Analysis; Secondary Research Question, Manipulation Check, Study 1: Mixed effects logistic regression results predicting whether participants selected a lower-energy option. 12](#_Toc87386394)

[Table S9. Descriptives: Proportion of selections by order of preference ranking, whether selected product was higher or lower energy, availability condition, food type and study 13](#_Toc87386395)

[Table S10a. Descriptives: Proportion of lower-energy product selections, by education 14](#_Toc87386396)

[Table S10b. Descriptives: Mean ranking of lower-energy and higher-energy options, by education 14](#_Toc87386397)

[Table S10c. Descriptives: Proportion of highest-ranked options that are lower-energy, by education 15](#_Toc87386398)

[Table S10d. Descriptives: Proportion of product selections by ranking, by education 16](#_Toc87386399)

# Analysis Plan: Secondary Research Questions

*Lower-energy vs. higher-energy highest-ranked* *product*:

*Hypothesis 2:* Analysed using mixed effects logistic regression, conducted at the participant-level, examining whether a dummy variable indicating whether participants’ highest-ranked option was lower energy vs. higher energy from ranges of options of (a) one lower-energy, three higher-energy options or (b) three lower-energy, one higher-energy options [for (i) branded snack or (ii) unbranded main meal options] alters the likelihood that participants select their highest-ranked option, with random effects for participant. Covariates included age, gender, and hunger.

*Food type analysis*:

*Hypothesis 1:* Analysis as for Research Question 1, including interactions between availability conditions and food type.

*Hypothesis 2:* Analysis as for whether participants’ highest-ranked option was lower energy vs. higher energy (above)highest-ranked, including interactions between availability conditions and food type.

*Socioeconomic analysis:*

*Hypothesis 1:* Analysis as for Research Question 1, including a variable for education.

*Hypothesis 2:* Analysis as for whether participants’ highest-ranked option was lower energy vs. higher energy highest-ranked, including interactions between availability conditions and education.

*N.B.* These analyses were dependent on a difference in response to the availability manipulation being observed by education.

# Table S1a. Manipulation check, Study 1: Mixed effects logistic regression results predicting whether participants selected a lower-energy option.

|  | **Odds ratio** | **95% CI** | | **p-value** |
| --- | --- | --- | --- | --- |
|  |  | **Lower** | **Higher** |  |
| Predominantly lower-energy availability (Ref: Predominantly higher-energy) | 8.9 | 7.9 | 10.1 | <0.001 |
| Snack (Ref: Main meal) | 1.3 | 1.1 | 1.4 | <0.001 |
| Age | 1.02 | 1.01 | 1.02 | <0.001 |
| Female (Ref: Male) | 1.2 | 1.1 | 1.4 | 0.001 |
| Hunger | 0.92 | 0.88 | 0.97 | 0.001 |
| Constant | 0.08 | 0.05 | 0.11 | <0.001 |
| Random effects: ID | 0.31 | 0.21 | 0.47 | - |

*7864 observations from 1966 participants: Two participants who reported gender as ‘Other’ were not included in analyses; Eight participants did not report age or hunger*

# Table S1b. Manipulation check, Study 2b: Mixed effects logistic regression results predicting whether participants selected a lower-energy option.

|  | **Odds ratio** | **95% CI** | | **p-value** |
| --- | --- | --- | --- | --- |
|  |  | **Lower** | **Higher** |  |
| Predominantly lower-energy availability (Ref: Predominantly higher-energy) | 9.7 | 7.0 | 13.5 | <0.001 |
| Age | 0.995 | 0.987 | 1.004 | 0.265 |
| Female (Ref: Male) | 1.5 | 1.1 | 2.0 | 0.005 |
| Hunger | 0.94 | 0.84 | 1.04 | 0.197 |
| Constant | 0.07 | 0.04 | 0.13 | <0.001 |
| Random effects: ID | 1.11 | 0.57 | 2.14 | - |

*2156 observations from 1078 participants*

# Table S2a. Research Question 1, Study 1: Mixed effects logistic regression results predicting whether participants’ highest-ranked option was a lower-energy option.

|  | **Odds ratio** | **95% CI** | | **p-value** |
| --- | --- | --- | --- | --- |
|  |  | **Lower** | **Higher** |  |
| Predominantly lower-energy availability (Ref: Predominantly higher-energy) | 10.1 | 8.9 | 11.4 | <0.001 |
| Snack (Ref: Main meal) | 1.3 | 1.2 | 1.5 | <0.001 |
| Age | 1.02 | 1.02 | 1.03 | <0.001 |
| Female (Ref: Male) | 1.3 | 1.1 | 1.4 | <0.001 |
| Hunger | 0.92 | 0.88 | 0.97 | 0.001 |
| Constant | 0.06 | 0.04 | 0.08 | <0.001 |
| Random effects: ID | 0.30 | 0.19 | 0.47 | - |

*7616 observations from 1966 participants: Two participants who reported gender as ‘Other’ were not included in analyses; Eight participants did not report age or hunger. Trials with a lower-energy and a higher-energy option tied for highest-ranked were excluded.*

# Table S2b. Research Question 1, Study 2b: Mixed effects logistic regression results predicting whether participants’ highest-ranked option was a lower-energy option

|  | **Odds ratio** | **95% CI** | | **p-value** |
| --- | --- | --- | --- | --- |
|  |  | **Lower** | **Higher** |  |
| Predominantly lower-energy availability (Ref: Predominantly higher-energy) | 10.4 | 7.4 | 14.7 | <0.001 |
| Age | 0.994 | 0.986 | 1.003 | 0.186 |
| Female (Ref: Male) | 1.5 | 1.2 | 1.97 | 0.007 |
| Hunger | 0.96 | 0.86 | 1.07 | 0.441 |
| Constant | 0.07 | 0.04 | 0.12 | <0.001 |
| Random effects: ID | 1.12 | 0.56 | 2.25 | - |

*2087 observations from 1076 participants. Trials with a lower-energy and a higher-energy option tied for highest-ranked were excluded.*

# Table S3. Secondary Research Question 3a, Study 1: Mixed effects logistic regression results predicting whether participants’ highest-ranked option was a lower-energy option.

|  | **Odds ratio** | **95% CI** | | **p-value** |
| --- | --- | --- | --- | --- |
|  |  | **Lower** | **Higher** |  |
| Predominantly lower-energy availability (Ref: Predominantly higher-energy) | 11.0 | 9.3 | 13.0 | <0.001 |
| Snack (Ref: Main meal) | 1.5 | 1.2 | 1.7 | <0.001 |
| Snack * Predominantly lower-energy availability | 0.8 | 0.7 | 1.0 | 0.123 |
| Age | 1.02 | 1.02 | 1.03 | <0.001 |
| Female (Ref: Male) | 1.3 | 1.1 | 1.4 | <0.001 |
| Hunger | 0.92 | 0.88 | 0.97 | 0.001 |
| Constant | 0.05 | 0.04 | 0.08 | <0.001 |
| Random effects: ID | 0.30 | 0.19 | 0.47 | - |

*7616 observations from 1966 participants: Two participants who reported gender as ‘Other’ were not included in analyses; Eight participants did not report age or hunger. Trials with a lower-energy and a higher-energy option tied for highest-ranked were excluded.*

# Table S4a. Secondary Research Question 4a, Study 1: Mixed effects logistic regression results predicting whether participants’ selected option corresponds to their highest-ranked option.

|  | **Odds ratio** | **95% CI** | | **p-value** |
| --- | --- | --- | --- | --- |
|  |  | **Lower** | **Higher** |  |
| Highest-ranked option is lower-energy (Ref: Higher-energy) | 0.80 | 0.67 | 0.95 | 0.013 |
| Predominantly lower-energy availability (Ref: Predominantly higher-energy) | 1.07 | 0.90 | 1.27 | 0.441 |
| Snack (Ref: Main meal) | 0.74 | 0.64 | 0.86 | <0.001 |
| Age | 1.01 | 1.00 | 1.01 | 0.046 |
| Female (Ref: Male) | 1.0 | 0.89 | 1.23 | 0.594 |
| Hunger | 1.05 | 0.99 | 1.12 | 0.121 |
| Constant | 7.64 | 4.67 | 12.49 | <0.001 |
| Random effects: ID | 0.40 | 0.22 | 0.72 | - |

*7338 observations from 1966 participants: Two participants who reported gender as ‘Other’ were not included in analyses; Eight participants did not report age or hunger. Trials with tied options for highest-ranked were excluded.*

# Table S4b. Secondary Research Question 4a, Study 2b: Mixed effects logistic regression results predicting whether participants’ selected option corresponds to their highest-ranked option.

|  | **Odds ratio** | **95% CI** | | **p-value** |
| --- | --- | --- | --- | --- |
|  |  | **Lower** | **Higher** |  |
| Highest-ranked option is lower-energy (Ref: Higher-energy) | 0.27 | 0.17 | 0.42 | <0.001 |
| Predominantly lower-energy availability (Ref: Predominantly higher-energy) | 2.0 | 1.4 | 2.9 | <0.001 |
| Age | 1.02 | 1.01 | 1.03 | 0.003 |
| Female (Ref: Male) | 0.88 | 0.58 | 1.33 | 0.551 |
| Hunger | 0.89 | 0.77 | 1.04 | 0.141 |
| Constant | 7.64 | 3.49 | 16.74 | <0.001 |
| Random effects: ID | 2.83 | 1.63 | 4.93 | - |

*1997 observations from 1071 participants (trials with tied options for highest-ranked were excluded)*

# Table S6a. Secondary Research Question, Manipulation Check, Study 1: Mixed effects logistic regression results predicting whether participants selected a lower-energy option.

|  | **Odds ratio** | **95% CI** | | **p-value** |
| --- | --- | --- | --- | --- |
|  |  | **Lower** | **Higher** |  |
| Predominantly lower-energy availability (Ref: Predominantly higher-energy) | 8.9 | 7.6 | 10.4 | <0.001 |
| Higher education (Ref: Lower education) | 1.1 | 0.9 | 1.3 | 0.222 |
| Predominantly lower-energy availability * Higher education | 1.0 | 0.8 | 1.2 | 0.893 |
| Snack (Ref: Main meal) | 1.3 | 1.1 | 1.4 | <0.001 |
| Age | 1.02 | 1.01 | 1.02 | <0.001 |
| Female (Ref: Male) | 1.2 | 1.1 | 1.4 | 0.001 |
| Hunger | 0.93 | 0.88 | 0.97 | 0.001 |
| Constant | 0.07 | 0.05 | 0.10 | <0.001 |
| Random effects: ID | 0.31 | 0.20 | 0.47 | - |

*7864 observations from 1966 participants: Two participants who reported gender as ‘Other’ were not included in analyses; Eight participants did not report age or hunger.*

# Table S6b. Secondary Research Question, Manipulation Check, Study 2b: Mixed effects logistic regression results predicting whether participants selected a lower-energy option.

|  | **Odds ratio** | **95% CI** | | **p-value** |
| --- | --- | --- | --- | --- |
|  |  | **Lower** | **Higher** |  |
| Predominantly lower-energy availability (Ref: Predominantly higher-energy) | 8.6 | 5.7 | 13.1 | <0.001 |
| Higher education (Ref: Lower education) | 1.4 | 0.9 | 2.1 | 0.189 |
| Predominantly lower-energy availability * Higher education | 1.3 | 0.8 | 2.1 | 0.381 |
| Age | 0.996 | 0.988 | 1.005 | 0.401 |
| Female (Ref: Male) | 1.5 | 1.2 | 2.0 | 0.003 |
| Hunger | 0.94 | 0.85 | 1.04 | 0.208 |
| Constant | 0.06 | 0.03 | 0.11 | <0.001 |
| Random effects: ID | 1.07 | 0.54 | 2.10 | - |

*2156 observations from 1078 participants*

# Table S7a. Income Group Analysis; Secondary Research Question, Manipulation Check, Study 1: Mixed effects logistic regression results predicting whether participants selected a lower-energy option.

|  | **Odds ratio** | **95% CI** | | **p-value** |
| --- | --- | --- | --- | --- |
|  |  | **Lower** | **Higher** |  |
| Predominantly lower-energy availability (Ref: Predominantly higher-energy) | 8.6 | 6.9 | 10.6 | <0.001 |
| Income: £17,500-£29,999 (Ref: Under £17,500) | 0.9 | 0.8 | 1.2 | 0.602 |
| Income: £30,000-£49,999 (Ref: Under £17,500) | 1.0 | 0.8 | 1.3 | 0.845 |
| Income: £50,000+ (Ref: Under £17,500) | 0.9 | 0.7 | 1.2 | 0.665 |
| Predominantly lower-energy availability * Income £17,500-£29,999 | 1.1 | 0.8 | 1.5 | 0.478 |
| Predominantly lower-energy availability * Income £30,000-£49,999 | 1.0 | 0.8 | 1.3 | 0.959 |
| Predominantly lower-energy availability * Income £50,000+ | 1.0 | 0.7 | 1.4 | 0.920 |
| Snack (Ref: Main meal) | 1.3 | 1.1 | 1.4 | <0.001 |
| Age | 1.02 | 1.01 | 1.02 | <0.001 |
| Female (Ref: Male) | 1.2 | 1.1 | 1.4 | 0.003 |
| Hunger | 0.92 | 0.88 | 0.97 | 0.001 |
| Constant | 0.08 | 0.05 | 0.12 | <0.001 |
| Random effects: ID | 0.31 | 0.20 | 0.47 | - |

*7424 observations from 1856 participants: Two participants who reported gender as ‘Other’ were not included in analyses; Eight participants did not report age, hunger or income group; 110 additional participants did not report income.*

# Table S7b. Income Group Analysis; Secondary Research Question, Manipulation Check, Study 2b: Mixed effects logistic regression results predicting whether participants selected a lower-energy option.

|  | **Odds ratio** | **95% CI** | | **p-value** |
| --- | --- | --- | --- | --- |
|  |  | **Lower** | **Higher** |  |
| Predominantly lower-energy availability (Ref: Predominantly higher-energy) | 13.3 | 7.3 | 24.4 | <0.001 |
| Income: £17,500-£29,999 (Ref: Under £17,500) | 1.5 | 0.8 | 3.1 | 0.220 |
| Income: £30,000-£49,999 (Ref: Under £17,500) | 1.3 | 0.7 | 2.6 | 0.396 |
| Income: £50,000+ (Ref: Under £17,500) | 1.6 | 0.8 | 3.2 | 0.183 |
| Predominantly lower-energy availability * Income £17,500-£29,999 | 0.4 | 0.2 | 0.9 | 0.019 |
| Predominantly lower-energy availability * Income £30,000-£49,999 | 1.0 | 0.5 | 2.0 | 0.895 |
| Predominantly lower-energy availability * Income £50,000+ | 0.7 | 0.3 | 1.6 | 0.389 |
| Age | 0.99 | 0.99 | 1.00 | 0.191 |
| Female (Ref: Male) | 1.6 | 1.2 | 2.1 | 0.002 |
| Hunger | 0.93 | 0.83 | 1.04 | 0.183 |
| Constant | 0.05 | 0.03 | 0.12 | <0.001 |
| Random effects: ID | 1.19 | 0.61 | 2.30 | - |

*2002 observations from 1001 participants; 77 participants did not report income*

# Table S8a. Occupational Group Analysis; Secondary Research Question, Manipulation Check, Study 1: Mixed effects logistic regression results predicting whether participants selected a lower-energy option.

|  | **Odds ratio** | **95% CI** | | **p-value** |
| --- | --- | --- | --- | --- |
|  |  | **Lower** | **Higher** |  |
| Predominantly lower-energy availability (Ref: Predominantly higher-energy) | 9.1 | 7.5 | 10.9 | <0.001 |
| Occupational group: C1&C2 (Ref: A&B) | 0.88 | 0.73 | 1.1 | 0.168 |
| Occupational group: D&E (Ref: A&B) | 1.0 | 0.8 | 1.3 | 0.845 |
| Predominantly lower-energy availability * C1&C2 | 1.1 | 0.8 | 1.4 | 0.560 |
| Predominantly lower-energy availability * D&E | 0.8 | 0.6 | 1.1 | 0.154 |
| Snack (Ref: Main meal) | 1.3 | 1.1 | 1.4 | <0.001 |
| Age | 1.02 | 1.01 | 1.02 | <0.001 |
| Female (Ref: Male) | 1.2 | 1.1 | 1.4 | 0.001 |
| Hunger | 0.93 | 0.88 | 0.97 | 0.001 |
| Constant | 0.08 | 0.05 | 0.11 | <0.001 |
| Random effects: ID | 0.31 | 0.20 | 0.47 | - |

*7840 observations from 1960 participants: Two participants who reported gender as ‘Other’ were not included in analyses; Eight participants did not report age or hunger; Occupational group was missing for six participants*

Occupational groups: A&B: Higher and intermediate managerial, administrative and professional occupations; C1&C2: Supervisory, clerical and junior managerial, administrative and professional occupations; D&E: Semi-skilled and unskilled manual occupations

# Table S9. Descriptives: Proportion of selections by order of preference ranking, whether selected product was higher or lower energy, availability condition, food type and study

|  | | | Proportion of product selections (% (n)) | | | | | | | |
| --- | --- | --- | --- | --- | --- | --- | --- | --- | --- | --- |
|  |  |  | Lower-energy product highest-ranked | | | | Higher-energy product highest-ranked | | | |
| *Order of preference ranking for option within range offered* | | | *1* | *2* | *3* | *4* | *1* | *2* | *3* | *4* |
| Study 1 | Branded snacks | Predominantly lower-energy availability | 87.1  (1223) | 11.0  (155) | 1.6  (23) | 0.2  (3) | 90.5  (456) | 8.5  (43) | 1.0  (5) | 0  (0) |
|  |  | Predominantly higher-energy availability | 89.2  (446) | 9.6  (48) | 1.2  (6) | 0  (0) | 89.0  (1257) | 9.9  (140) | 0.8  (12) | 0.2  (3) |
|  | Unbranded meals | Predominantly lower-energy availability | 91.0  (1216) | 8.0  (107) | 0.6  (8) | 0.4  (5) | 92.6  (538) | 6.5  (38) | 0.9  (5) | 0  (0) |
|  |  | Predominantly higher-energy availability | 88.7  (337) | 10.3  (39) | 1.1  (4) | 0  (0) | 91.5  (1406) | 7.3  (112) | 1.1  (17) | 0.1  (2) |
| Study 2b | Unbranded meals | Predominantly lower-energy availability | 80.7  (346) | 14.0  (60) | 3.5  (15) | 1.9  (8) | 93.7  (569) | 5.8  (35) | 0.3  (2) | 0.2  (1) |
|  |  | Predominantly higher-energy availability | 79.6  (74) | 11.8  (11) | 7.5  (7) | 1.1  (1) | 87.9  (842) | 9.2  (88) | 1.6  (15) | 1.4  (13) |

N.B. Trials with tied highest-ranked options that were lower-energy and higher-energy were excluded. Other ties (e.g. 1.5) were rounded down (i.e. 1.5 to 1).

# Table S10a. Descriptives: Proportion of lower-energy product selections, by education

|  | | Proportion of lower-energy product selections (% (n)) | | |
| --- | --- | --- | --- | --- |
|  |  | Study 1 | | Study 2b |
|  |  | Branded snacks | Unbranded meals | Unbranded meals |
| Predominantly lower-energy availability | Higher education | 72.0  (716) | 69.7  (693) | 47.2%  (254) |
|  | Lower education | 72.0  (707) | 67.8  (666) | 36.7%  (198) |
| Predominantly higher-energy availability | Higher education | 25.6  (254) | 23.1  (230) | 10.8%  (58) |
|  | Lower education | 28.0  (275) | 19.2  (189) | 8.3%  (45) |

# Table S10b. Descriptives: Mean ranking of lower-energy and higher-energy options, by education

|  | Mean ranking (s.d.) | | | | | |
| --- | --- | --- | --- | --- | --- | --- |
|  | Study 1 | | | | Study 2b | |
|  | Branded snacks | | Unbranded main meals | | Unbranded main meals | |
|  | Lower-energy items | Higher-energy items | Lower-energy items | Higher-energy items | Lower-energy items | Higher-energy items |
| Higher education | 4.40 (0.99) | 4.60 (0.99) | 4.57 (0.96) | 4.43 (0.96) | 5.40 (0.99) | 3.60 (0.99) |
| Lower education | 4.38 (0.98) | 4.62 (0.98) | 4.70 (0.94) | 4.30 (0.94) | 5.60 (0.91) | 3.40 (0.91) |
| Mann-Whitney U^1^ | Z=-0.218 (p=0.8277) | | Z=3.075 (p=0.0021) | | Z=3.451 (p=0.0006) | |

^1^ Test for differences by education

# Table S10c. Descriptives: Proportion of highest-ranked options that are lower-energy, by education

|  | | | Proportion of highest-ranked options that are lower-energy (% (n)) | |
| --- | --- | --- | --- | --- |
|  |  |  | Predominantly higher-energy range | Predominantly lower-energy range |
| Study 1 | Branded snacks | Higher education | 25.8 (247) | 72.9 (703) |
|  |  | Lower education | 26.5 (253) | 74.3 (701) |
|  | Unbranded meals | Higher education | 18.5 (178) | 71.2 (688) |
|  |  | Lower education | 21.1 (202) | 68.1 (648) |
| Study 2b | Unbranded meals | Higher education | 9.9 (52) | 46.8 (239) |
|  |  | Lower education | 7.8 (41) | 36.2 (190) |

N.B. Trials with tied highest-ranked options that were lower-energy and higher-energy were excluded

# Table S10d. Descriptives: Proportion of product selections by ranking, by education

|  | | | Proportion of product selections (% (n)) | | | | | | | |
| --- | --- | --- | --- | --- | --- | --- | --- | --- | --- | --- |
|  |  |  | Higher education | | | | Lower education | | | |
| Order of preference ranking for option within range offered | | | 1 | 2 | 3 | 4 | 1 | 2 | 3 | 4 |
| Study 1 | Branded snacks | Predominantly lower-energy availability | 88.6  (881) | 9.7  (96) | 1.5  (15) | 0.2  (2) | 87.8  (862) | 10.7  (105) | 1.3  (13) | 0.2  (2) |
|  |  | Predominantly higher-energy availability | 88.6  (881) | 10.2  (101) | 1.0  (10) | 0.2  (2) | 90.1  (885) | 8.9  (87) | 0.9  (9) | 0.1  (1) |
|  | Unbranded meals | Predominantly lower-energy availability | 91.9  (913) | 7.3  (73) | 0.5  (5) | 0.3  (3) | 91.3  (897) | 7.5  (74) | 0.9  (9) | 0.2  (2) |
|  |  | Predominantly higher-energy availability | 90.9  (903) | 7.7  (76) | 1.2  (12) | 0.2  (2) | 91.3  (897) | 7.6  (75) | 10.0  (10) | 0  (0) |
| Study 2b | Unbranded meals | Predominantly lower-energy availability | 86.8  (467) | 9.9  (53) | 1.7  (9) | 1.7  (9) | 90.2  (487) | 8.3  (45) | 1.5  (8) | 0  (0) |
|  |  | Predominantly higher-energy availability | 84.9  (457) | 10.0  (54) | 2.8  (15) | 2.2  (12) | 89.1  (481) | 8.3  (45) | 1.5  (8) | 1.1  (6) |

N.B. Ties (e.g. 1.5) were rounded down (i.e. 1.5 to 1)
